# An Ethics Framework for Evaluating Ownership Practices in Biomedical Citizen Science

**DOI:** 10.5334/cstp.537

**Published:** 2022-12-15

**Authors:** CHRISTI J. GUERRINI, AMY L. MCGUIRE

**Affiliations:** Baylor College of Medicine, US; Baylor College of Medicine, US

**Keywords:** Ownership, IRB, ethics, Common Rule, biomedical, health

## Abstract

The collaborative nature of citizen science raises important questions about managing ownership of its research outputs. Potential citizen science research outputs include data sets, findings, publications, and discoveries of new ideas, methods, products, and technologies. Unlike citizen science projects conducted in other disciplines, biomedical citizen science projects often include features, such as contribution of personal health data, that might heighten citizen scientists’ expectations that they will be able to access, control, or share in the benefits of project outputs. Here, we refer to moral claims of access, control, and benefit as ownership claims, and a project’s management of ownership claims as its ownership practices. Ethical management of ownership is widely recognized as an important consideration for citizen science projects, and practitioners and scholars have described helpful recommendations for preempting issues and engaging stakeholders on practices. Building on this literature, we propose a framework to help biomedical citizen science projects systematically evaluate the ethical soundness of their ownership practices based on four considerations: reciprocal treatment, relative treatment, risk-benefit assessment, and reasonable expectations.

## BACKGROUND

**Figure 1:**
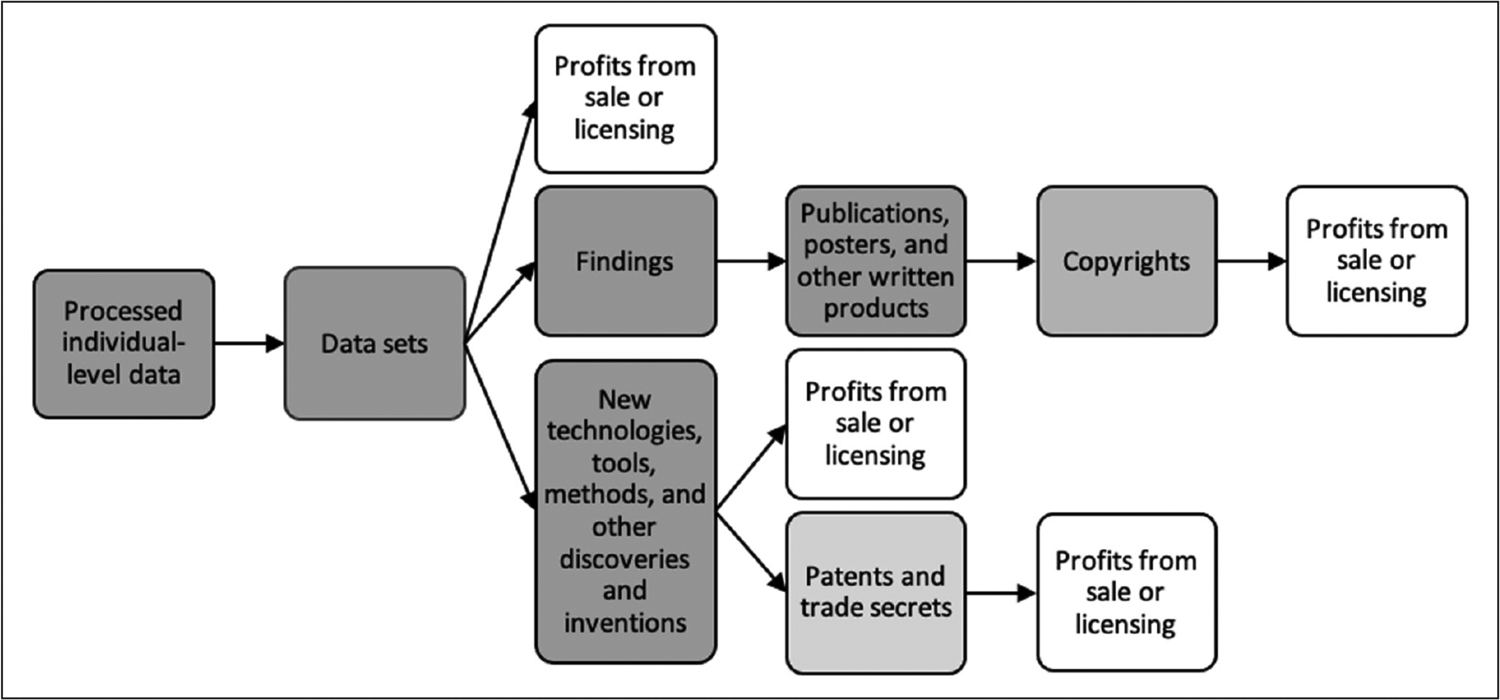
Potential outputs of citizen science projects. Data = information recorded in any format or medium. Arrows show that outputs can generate or lead to other outputs.

Ownership of research outputs has been identified as an important practical, legal, and ethical issue for many citizen science projects (Dratwa 2015; Fiske, Prainsack, and Buyx 2019; Guerrini et al. 2018; Resnik, Elliott, and Miller 2015; Riesch and Potter 2014; Scassa and Chung 2015a; Vayena and Tasioulas 2015). Whereas project inputs include contributions to a project, such as data and biospecimens, outputs are the tangible and intangible ideas and things that result from research processes. Depending on the design and objectives of a project, its outputs might include data sets, research findings, publications, or discoveries of new methods, products, or technologies (Figure 1).

Although citizen scientists might not have property rights to project outputs, factors internal and external to a project can promote the idea that they have valid claims to access, control, or share in the benefits of the project’s outputs. This second, more expansive, meaning of ownership claims—as claims grounded in ethical principles, whether or not recognized as rights secured by law (Guerrini et al. 2019)—is the focus of this article. More specifically, and in recognition of the fact that some projects are led by or in partnership with citizen scientists, it is concerned with the ownership claims of citizen scientists who are not project leaders. Further, this article is focused on ownership of project outputs, not inputs. Nevertheless, literature and legal precedent considering the propertization and commercialization of personal research inputs (Roberts 2017; Roberts, Pereira, and McGuire 2017; Spector-Bagdady et al. 2018) are relevant to the extent that persistent rights in inputs influence moral claims to ownership of outputs.

Project practices that facilitate citizen scientists’ exclusive or non-exclusive access to, control of, or share in the benefits of research outputs are referred to as ownership practices. Examples of ownership practices include providing citizen scientists access to processed individual-level data and data sets; coauthoring publications with citizen scientists; sharing with citizen scientists the profits from commercial transactions and royalties from published works; inviting citizen scientists to co-present results and sharing honoraria from speaking engagements; recognizing or securing legal rights of citizen scientists to intellectual property (IP); and facilitating citizen scientists’ access to commercialized products or technologies that result from their work. A project might, but need not, document or formalize these practices in public statements, policies, or agreements.

Unlike citizen science projects conducted in other disciplines, such as ecology and space science, biomedical citizen science projects sometimes include features that might be especially likely to stir moral claims of ownership. Although biomedical citizen science projects are diverse (Table 1), a common feature is their invitation for participants to contribute personal health data or biospecimens for analysis (ECSA 2020; Fiske, Prainsack, and Buyx 2019; Wiggins and Wilbanks 2019). Depending on the circumstances, the intimate nature of these inputs can prompt feelings of ownership over them that extend to the outputs that result from analysis and use of those inputs. Genetic and genomic data, including data generated from single nucleotide polymorphism (SNP) microarrays and whole genome and exome sequencing, are arguably some of the most personal kinds of biomedical data that a person can share given that they can be predictive of individual health, behavior, and appearance. Separately, participants might have heightened feelings of entitlement to access or use biomedical citizen science research outputs when projects focus on medical conditions that affect them or their loved ones and they stand to benefit from, or might be harmed by, those outputs.

Empirical studies have described citizen science project practices related to accessing, controlling, and sharing in the benefits of outputs, with a focus on data sets, and probed relevant perspectives of citizen science practitioners, participants, and scholars (Borda, Gray, and Fu 2019; Bowser et al. 2020; Guerrini et al. 2019; Guerrini et al. 2022). Potential legal claims of citizen scientists to project outputs also have been examined (Guerrini and Contreras 2020; Scassa and Chung 2015a,b).

In these and other publications, ethical management of ownership is widely recognized as an important consideration for citizen science projects. For example, the European Citizen Science Association (2020) describes the practice of informing citizen scientists how their data are used (such as by sharing outputs with citizen scientists who contributed to them) as an ethical responsibility of projects. Focusing on IP, Scassa and Chung (2015a, p. 5) explain: “IP management in citizen science has ethical dimensions because IP rights regulate a series of relationships between individuals and in relation to intangible goods.” Especially when collaborators are in a hierarchical relationship, IP management “must be carried out ethically to sustain trust” between them (Scassa and Chung 2015a, p. 5). In the context of ownership of results of participatory health research and development, an advisory body of the President of the European Commission makes an explicit appeal to fairness in asking whether “greater reflection [should] be paid to allowing citizens/patients to share in the advantages of their contributions” when later monetized by others (Dratwa 2015, p. 60).

To assist in management of research outputs, scholars have identified ethical issues to consider when making decisions related to access to data and data sets (Cooper, Rasmussen, and Jones 2021) and sharing benefits of commercial exploitation, including royalties in inventions, with participant communities and individual citizen scientists (Scassa and Chung 2015a). They have also provided useful suggestions for management of ownership that include negotiating instruments “that recognize the interests of all stakeholders” and discussing ownership and IP with citizen scientists at the beginning of and as needed during the project period to promote understanding (Resnik, Elliott, and Miller 2015, p. 478).

Building on this literature, we propose a framework to help biomedical citizen science project leaders systematically evaluate the ethical soundness of ownership practices they are considering or have implemented. We begin by describing the traditional approach to ethical review of scientific research, including its ownership-relevant plans. We then explain why the traditional approach does not always translate well to scientific activities designed as citizen science, necessitating consideration of a different approach to ethical review of their ownership practices. In an effort to meet that challenge, we propose a framework for evaluating those practices that centers on four considerations: reciprocal treatment, relative treatment, risk-benefit assessment, and reasonable expectations. Although this framework might be useful to projects unrelated to health or medicine, we focus its application on biomedical projects given the special issues they raise related to ownership, as described above. Accordingly, we anchor the discussion with a hypothetical project relevant to human genetics and genomics, a popular focus of self-inquiry (White 2019). Ultimately, the intention of the proposed “4R” framework is to help citizen science projects evaluate the ethical defensibility of their ownership practices.

## A HYPOTHETICAL

Consider a hypothetical citizen science project called PersonalPRS. The PRS in the project’s title refers to polygenic risk score, which is an estimate of a person’s risk for a disease based on the combination of their genetic variants (Torkamani, Wineinger, and Topol 2018). The aims of the project, run by a small group of United States (US)–based Ph.D. students during their free time, are twofold: (1) most immediately, to generate PRS for common diseases for individuals from their SNP files, and (2), for specific diseases, to identify environmental exposures common among individuals who have the disease but low PRS for it.

Individuals can contribute to the project in several ways. First, they are invited to upload their SNP files, previously obtained from direct-to-consumer (DTC) genetic testing companies, and complete a brief questionnaire about their health history. The crowdsourced data are used to improve PRS construction methods. In return, data contributors are provided their PRS for common diseases. Second, in an online forum, individuals (including but not limited to data contributors) are invited to suggest modifications to the project’s PRS construction methods and calculations, which are published on the platform. Elsewhere in that forum, third, they are invited to discuss their PRS results and suggest improvements to the platform and its communication of results, some of which the project leaders have implemented. The leaders have invited the most active participants—called SuPRStars—to serve as moderators of the forum. SuPRStars are in regular, direct contact (by email and video conference) with the project leaders.

With respect to future plans, the project leaders hope to roll out disease-specific challenges in which self-selecting participants will work to identify environmental exposures common among individuals who have a specific disease but low PRS for it. Specifically, participants will partner with project leaders to develop supplemental questionnaires that will be sent to these individuals and analyze deidentified SNP files linked to deidentified questionnaire responses contributed by participants who have specifically opted into this research use of their data.

The PersonalPRS website describes the project as “citizen science” and invites members of the public to “join the team” as “partners.” The website also includes a disclaimer that the platform is provided for educational purposes only; is not intended to be used for any diagnostic, treatment, or preventative purpose; and is not a substitute for professional medical advice.

Approximately 75,000 individuals around the world have uploaded their SNP files to PersonalPRS and completed health questionnaires. Recently, the leaders were approached by a DTC genetic testing company interested in purchasing the PersonalPRS platform and participants’ deidentified SNP files linked to their deidentified questionnaire responses.

## THE TRADITIONAL RESEARCH ETHICS FRAMEWORK AND APPLICATION TO OWNERSHIP PRACTICES

There are several ways that PersonalPRS participants can engage with the platform, with project leaders, and with each other. Each of these interactions generates distinct research outputs. What criteria should be used to evaluate the ethical acceptability of approaches to manage claims to access, control, and share in the benefits of those outputs?

According to a traditional research ethics analysis, this question would be answered primarily by reference to the principles identified in the Belmont Report, which is the basis of ethical oversight of biomedical research provided by Institutional Review Boards (IRBs) in the US (National Commission 1979). Briefly, there are two sets of federal regulations that require IRB review of US-based biomedical research. First, the Federal Policy for the Protection of Human Research Subjects, otherwise known as the Common Rule, applies to all non-exempt research involving human subjects conducted or funded by the US federal government (45 C.F.R. § 46). Like many citizen science projects, PersonalPRS is probably not subject to the Common Rule’s IRB requirements because its activities are not conducted or funded by the federal government and, to the extent that any of the leaders’ institutions apply the Common Rule to all human subjects research in which they are engaged as a matter of institutional policy (Meyer 2020), the institutions do not appear to have sufficient nexus to project activities to be within their IRBs’ jurisdiction.

The US Food and Drug Administration (FDA) requires IRB review of certain clinical investigations involving FDA-regulated medical devices, including software that functions as a medical device, and these rules largely mirror the Common Rule’s procedural and substantive details (21 C.F.R. §§ 50, 56). There is a question whether any of PersonalPRS’s activities qualify as a clinical investigation requiring IRB review under FDA rules, but that question is moot if PersonalPRS does not qualify as an FDA-regulated medical device. The FDA has generally ignored third-party genetic interpretation platforms (Greely 2020), many of which include disclaimers similar to those published on the PersonalPRS platform, suggesting that the agency might view them as the kind of low-risk device over which it has stated it will exercise enforcement discretion even if subject to FDA regulations (FDA 2019a,b).

Still, some US states extend federal research subject protections to research over which it has jurisdiction, and many journals require IRB approval of research that they publish (Meyer 2020; Resnik 2019). For these or other reasons, some otherwise unregulated research, including citizen science projects, might seek out IRB review by commercial boards when they can afford it.

If an IRB reviewed the activities of PersonalPRS, whether such review is required or requested, the activities would be scrutinized using a lens focused on the three ethical principles outlined in the Belmont Report. First, respect for persons encompasses respect for their autonomy, or ability to make considered decisions for themselves (National Commission 1979). As operationalized by the federal rules (45 C.F.R. §§ 46.111(a)(4), 116; 21 C.F.R. §§ 50.20, 50.25, 56.111(a)(4)), demonstrating respect for research participants means obtaining their voluntary and informed consent to participate in research based on a clear articulation of the research aims and procedures, its anticipated risks and benefits, and alternatives to participation. Second, beneficence is an obligation to secure the well-being of research participants through attention to the risks and benefits of research activities (National Commission 1979). Risks and benefits can be physical, psychological, social, economic, or legal in nature. According to the federal rules, and representing a departure from the Belmont Report (Meyer 2020), risks must be minimized and reasonable in relation to benefits to participants and knowledge to be gained, but benefits need not be maximized (45 C.F.R. § 46.111(a) (1)-(2); 21 C.F.R. § 56.111(a)(1)-(2)). Third, justice in traditional research ethics is focused on fair distribution of the benefits and burdens of research, which can be evaluated according to formulations such as “to each person an equal share” or “to each person according to individual effort” (National Commission 1979). Both the Belmont Report and federal rules operationalize justice as fair selection of participants, including consideration of whether they belong to groups who are likely to benefit from research outputs, such as improved treatments (45 C.F.R. § 46.111(a)(3); 21 C.F.R. § 56.111(a)(3); National Commission 1979).

Neither the Common Rule nor FDA regulations require that researchers adopt practices to facilitate research participants’ access to, control of, or share in the benefits of research outputs, including data, findings, treatments, or technologies. Rather, the rules focus on making relevant plans transparent to research participants. Thus, in certain circumstances, the regulations require that prospective participants be informed if “clinically relevant research results, including individual research results, will be disclosed” to them (45 C.F.R. § 46.116(c)(8)). (This is distinct from the statement, required in certain circumstances, that “significant new findings” will be provided to participants that might relate to their willingness to continue participation (45 C.F.R. § 46.116(c)(5); 21 C.F.R. § 50.25(b) (5)).) Further, prospective participants must be informed if their biospecimens “may be used for commercial profit” and whether they will share in those profits (45 C.F.R. § 46.116(c)(7)). According to the federal agencies that have adopted these rules, informing prospective participants how their inputs will be used promotes their autonomy (HHS 2017).

Yet, in the absence of regulatory obligations to do so, some researchers have voluntarily adopted practices that recognize participants’ interests in the outputs of the work they support. For example, some have adopted plans to return individual-level data, such as raw genetic data files, to participants who indicated they would like this information (Thorogood et al. 2018). (This practice is distinct from the federal obligation of covered entities, including some researchers, to provide patients their protected health information on request (45 C.F.R. § 164.524).) These plans are justified on grounds that they promote autonomy and beneficence with respect to participants who are interested in knowing this information and potentially using it for their own benefit or sharing it to benefit others. However, as one scholar has noted, the Common Rule “clearly perceives return of individual results to be a source of potential harm to participants” (Meyer 2020, p. 68). For this reason, IRBs might scrutinize plans to return individual results to ensure that their perceived risks— for example, that participants could misunderstand the results and change their behaviors in potentially harmful ways based on the misunderstanding—are minimized. Ultimately, IRBs can decline to approve such plans.

But citizen scientists have a different role in the research process than participants in traditional biomedical research, prompting some scholars to examine whether conventional ethical frameworks are appropriate and sufficient for biomedical citizen science (Kasperowski, Hagen, and Rohden 2021; Rasmussen 2021; Vayena and Tasioulas 2013). Specifically, citizen scientists make research contributions other than or in addition to serving as a source of specimens and data for analysis by others and, as demonstrated by PersonalPRS, include potential contributions at many stages of the research process (ECSA 2015; Fiske et al. 2019; Wiggins and Wilbanks 2019). Consistent with these diverse and potentially robust participatory opportunities, some citizen science projects are structured to share power and decision-making with participants (Rasmussen 2019). Such projects might describe their objectives and the role of citizen scientists in relevant activities using a rhetoric of engagement and democratization (Fiske, Prainsack, and Buyx 2019) that could be inappropriate in communications about traditional biomedical research (Woolley et al. 2016).

Analysis of the ethical defensibility of ownership practices of biomedical citizen science projects requires an approach that takes into account these departures from traditional research. For example, depending on the project, respect for persons might require ongoing transparency about elements of projects that are not usually shared with traditional research participants, such as reporting data and findings in ways that will allow citizen scientists to “make meaning” of them (Cooper, Rasmussen, and Jones 2021, p. 2). Further, citizen scientists are sometimes in need of protection against different kinds of harms than traditional research participants (Rasmussen 2021). Some protections, such as prohibitions on the return of interpretations of contributed data, might be viewed by citizen scientists as paternalistic. They might also be confusing and disappointing given that, as noted by Wolf (2020, p. 163), return of results is a “core element[]” of participant-driven forms of citizen science. As a final example, justice concerns shift to providing fair opportunity to participate and minimizing barriers to participation, consistent with citizen science’s focus on inclusivity (Wiggins and Wilbanks 2019), and also encompasses fair distribution of opportunities to access, control, or share in the benefits of research outputs.

## THE 4R FRAMEWORK

For all of these reasons, citizen science projects are advised to give careful attention to ownership practices. Yet, there does not exist practical guidance for project leaders or external reviewers, such as IRBs, community ethics committees, or ethics consultants, regarding how to systematically evaluate the ethical soundness of practices that citizen science projects are considering or have adopted.

To address this gap, we propose a framework for evaluating ownership practices in biomedical citizen science comprising four considerations: reciprocal treatment, relative treatment, risk-benefit assessment, and reasonable expectations (Table 2). As described below, the considerations are rooted in traditional research ethics principles of respect for persons, beneficence, and justice, but consistent with the literature, their application pays special attention to the emphasis in citizen science on participatory experience, inclusivity, balance of power, and trust (Chesser, Porter, and Tuckett 2020; Fiske, Prainsack, and Buyx 2019; Groot and Abma 2022; Rasmussen 2019, 2021; Wiggins and Wilbanks 2019).

Importantly, the framework describes a standardized process for evaluating the ethical soundness of ownership practices. It does not describe a formula that will generate an absolute truth as to the rightness (or not) of any particular practice. However, by providing a guide for conducting these evaluations, the framework can help ensure that they are comprehensive and illuminate ways that a project might modify its practices to improve their ethical defensibility.

The intended users of the framework are project leaders and external reviewers, although our description focuses on evaluation by project leaders given that external review can be difficult to secure. For some projects, leaders might be difficult to identify; governance might be distributed among all or many participants. Usually, however, the leaders are a subset of the project’s institution-based scientists and/or citizen scientists, or if the project does not involve institution-based scientists, they are a subset of the project’s citizen scientists. We return to questions of responsibility and accountability for evaluation in the Discussion.

### RECIPROCAL TREATMENT

The first two considerations are grounded in the ethical principle of justice. First, reciprocal treatment considers whether a specific practice related to citizen scientists’ ownership of research outputs is fair given the quantity and/or quality of their research inputs. So conceptualized, this consideration is consistent with conceptions of distributive justice that what one is owed depends on their effort or contributions.

We emphasize that it is not our intention to frame the relationship between projects and citizen scientists as purely or even primarily transactional. Like traditional biomedical research participants, many biomedical citizen scientists likely have altruistic reasons for joining projects—namely, to advance scientific knowledge and to improve the health of individuals and populations (Curtis 2015; Damiani et al. 2021; Del Savio, Prainsack, and Buyx 2017; Land-Zandstra et al. 2016). However, citizen scientists are not always compensated for their time or efforts, and unlike traditional studies, citizen science projects might even ask citizen scientists for financial contributions to sustain them (Fiske, Prainsack, and Buyx 2019). It therefore seems especially important for projects to recognize their contributions in other ways (Chesser, Porter, and Tuckett 2020). While the full inventory of potential opportunities available to a project depends on its particulars, we suspect that most projects will be able to reciprocate through practices that facilitate, in some form, citizen scientists’ access to, control of, or share in the benefits of research outputs.

Projects are not ethically required to implement the most generous ownership practices. Instead, they should aim to meet, with respect to each citizen scientist, a minimum standard of fairness given the quantity and/or quality of their specific inputs. This step therefore requires careful delineation of those inputs. What exactly did the citizen scientist do in service to the project? How frequent were the contributions, and over what period of time, and what effort was required to make them? How significant were the contributions to the success of the project?

This consideration does not specify a particular formulation for a fair distribution given the lack of consensus as to the best or optimal formulation, and depending on the circumstances, multiple formulations might be appropriate. However, we are optimistic that analysis of reciprocal treatment with respect to specific practices can occur without this specification. As observed by Millum (2012, p. 219) in the context of preventing exploitation in cross-national research, “[d]espite the absence of general principles for working out which distributions of benefits and burdens are fair, … people have strong intuitions about fairness in particular cases.”

Turning to the example of PersonalPRS, platform participants provide two kinds of inputs: they upload their SNP files and complete a health questionnaire. Both contributions occur at one point in time—they are not ongoing—and can be completed in, let’s say, 15 minutes. Although each data file is a useful contribution to the project, the real value of these data comes from their combination into large-scale data sets. Given the low effort and significance associated with individual inputs, the practice of providing data contributors PRS for common diseases from their data likely meets minimum criteria for a fair exchange. We also note the symmetry between the nature of the inputs (raw data) and the outputs to which citizen scientists are provided access (interpretations of raw data), which is not necessary but might lend additional support to the conclusion that the practice satisfies minimum reciprocity.

If project leaders sell the PersonalPRS data set and platform, reciprocity alone will probably not harshly judge exclusion of data contributors from sharing in the profits. Again, reciprocity recognizes a lower bound on sharing in the benefits of research outputs with citizen scientists that projects are free to but need not exceed, where the location of that lower bound for each citizen scientist depends on what, exactly, they contributed to the project. Compared to data contributors, PersonalPRS participants who propose improvements to PRS construction methods or the project platform, whether in the online forum or in direct conversation with leaders, especially when those efforts are ongoing, time-intensive, and increase the scientific and social value of the project’s outputs, have a stronger ethical claim to profits from their sale. This example underscores that reciprocity is determined on an individualized basis and can support the implementation of different ownership practices for citizen scientists making different contributions to the same project.

### RELATIVE TREATMENT

Whereas reciprocal treatment is focused on the lower bound on sharing in the benefits of research outputs with citizen scientists, relative treatment is focused on the upper bound. Both are grounded in distributive justice concerns, but relative treatment shifts the perspective of concern. For reciprocal treatment, the question is narrow and individualized: does an ownership practice vis-à-vis each of a project’s citizen scientists satisfy minimum criteria for a fair exchange given their particular contributions? For relative treatment, the question is broad and relational: is a practice vis-à-vis some of a project’s citizen scientists too generous from the perspective of the project’s other citizen scientists given meaningful differences in their contributions?

Relative treatment undermines the ethical soundness of an ownership practice in two circumstances. The first is when the practice applies equally to citizen scientists who make unequal contributions. The practice might satisfy minimum criteria for a fair exchange and so not present lower bound problems. However, it might be too generous to some and so present upper bound problems. Returning to the sale of the PersonalPRS data set and platform, project leaders might have an upper bound problem if they provide both data contributors and SuPRStars an equal share in profits from the sale. From the perspective of the SuPRStars, this ownership practice might be unfair for failure to recognize that they made more, and more valuable, contributions to the project that assured its success.

The second circumstance when relative treatment applies is when an ownership practice results in disparate outcomes for a project’s citizen scientists that are justified by differences in their contributions, but the disparity itself is so large that it raises justice concerns. Let’s say that the purchaser of PersonalPRS is a leader in type II diabetes research and so is interested in the sale because it includes linked data and questionnaires from a large number of individuals reporting a type II diabetes diagnosis. Let’s also say that the project leaders plan to share the profits with participants but want to give a greater share to type II diabetic participants given that ownership of their information is most attractive to the purchaser. In this case, an unequal distribution of profits among the participants might be justified on reciprocal treatment grounds because of the difference in monetary value (from the perspective of the purchaser) in their individual inputs. However, depending on the magnitude of the inequality, the plan could raise relative treatment concerns. The project’s participants might be comfortable, for example, with a plan that gives type II diabetic participants two times the profit share of other participants, but what about thirty times? As we heard during interviews with genomic citizen science stakeholders (Guerrini et al. 2022), at a certain point, the difference becomes indefensible. Identifying that point is challenging and context dependent, but factors to consider include the distribution ratio (e.g., 1:2 v. 1:30) and absolute value of the benefits (e.g., $2 v. $4, $2,000 v. $60,000). Reasonable expectations, described below, are also relevant if citizen scientists are directly or indirectly led to believe that a project will place limits on disparities resulting from ownership practices.

### RISK-BENEFIT ASSESSMENT

Conducting a risk-benefit assessment is grounded in the ethical principle of beneficence. It asks how citizen scientists will be impacted by a particular ownership practice, and like regulatory frameworks for evaluation of medical research protocols, aims to minimize anticipated harms to participants associated with the practice. It also takes into account potential offsetting benefits.

So described, the analysis required by this consideration might look similar to the risk-benefit analyses conducted by IRBs reviewing medical research protocols, but there are important substantive and procedural differences. Assessment of harms and benefits of medical research requires methodological evaluation of the study design to ensure that it is scientifically sound and capable of producing socially valuable results; empirical judgments about the robustness and relevance of data regarding harms (usually framed as risks of harm given uncertainty) and benefits; and normative evaluation of the magnitude of those harms and benefits (Rid and Wendler 2011). Notably, compensation to participants is usually not considered a benefit in these assessments (HHS n.d.; Wertheimer 2013). Moreover, the risks of harm and benefits of therapeutic interventions are evaluated in comparison to alternative treatments (Rid and Wendler 2011).

Given the broad scope and comprehensiveness of analysis, evaluation of research risks and benefits is notoriously challenging. It also is outcome determinative. In the United States, if an IRB concludes that risks of harm to research subjects are not minimized and reasonable in relation to the benefits and the knowledge to be gained, it will not—indeed, it cannot—approve the study (45 C.F.R. § 46.111(a)(1)–(2); 21 C.F.R. § 56.111(a)(1)–(2)).

By contrast, the risks and benefits of this framework have a very narrow focus: they are concerned only with the anticipated outcomes of a project’s ownership practice. The analysis does not require comparison of the practice under review to alternative practices, and in many cases, it will not involve evaluation of any data (although it will include consideration of financial implications for citizen scientists). For these reasons, the analysis is simplified relative to traditional ethics review. Indeed, in some cases, an ownership practice may be associated only with potential benefits to a project’s citizen scientists or associated only with risks of harm to them. Examples of the former, assuming that research results are not stigmatizing (Guerrini and Contreras 2020), include a policy of publication in open-access journals and the creation and dissemination of non-specialist summaries and other lay-friendly products (Rasmussen 2021; Ross-Hellauer et al. 2020; Smith, Bélisle-Pipon, and Resnik 2019). An example of the latter, adopted from a lawsuit involving medical researchers (Greenberg v. Miami Children’s Hospital Research Institute 2003), is a practice denying citizen scientists access to a beneficial new medical technique or product that resulted from their contributions—for example, by patenting and then licensing the discovery to an entity that charges monopoly prices to access it. The practice would not appear to benefit anyone other than the patent holders and potentially would risk harm to citizen scientists if their health directly or indirectly depended on access to the discovery.

Risks of harm are not limited to physical harms but include, for example, considerations of implications for privacy concerns. This kind of harm might result if PersonalPRS published participants’ deidentified SNP files for reuse by other citizen scientists and researchers, following the model of openSNP, an online platform that crowdsources and publishes individual-level genetic and phenotypic data for secondary study (Greshake et al. 2014). It is possible that others might reidentify participants from their information and use it to embarrass, discriminate against, or otherwise harm them or their genetic relatives. One way to mitigate these harms is through robust disclosure of risks and adoption of procedures designed to ensure that participants understand and voluntarily accept them. As one model, the Personal Genome Project is a biomedical citizen science initiative that publishes deidentified whole genome sequencing data, genome reports, trait survey responses, and other health data of participants for secondary research use, and it describes these and other economic, legal, social, and dignitary harms that individuals could experience in a detailed consent form (PGP 2022). Another way to minimize harm is by allowing participants to opt out of public disclosure if it would not undermine project objectives to provide this opportunity.

Importantly, what qualifies as a harm or a benefit in this framework should account for the particular relationship between project leaders and non-leaders and their respective expertise, knowledge, inputs, responsibilities, and power. For example, an IRB’s concern that traditional research participants might misunderstand individual health-related research results if returned to them might not be a legitimate risk in the context of citizen science projects involving sophisticated volunteers. Risk tolerances may also be relevant. Focusing on tolerances related to privacy, Evans (2020, p. 80) observed: “Not all people value privacy as much as Institutional Review Boards and privacy advocates assume people do.”

Finally, and unlike traditional ethics review, consideration of the balance of risks and benefits of an ownership practice is not necessarily outcome determinative. As a practical matter, the practice might be necessary to ensure the long-term success of the project or to comply with contractual obligations. For example, obtaining and then licensing patents on project discoveries might be required by technology transfer agreements if a traditional scientific institution is involved. However, the ethical defensibility of this practice could be improved by clearly disclosing patenting intentions and obligations to citizen scientists at the time they join a project, thus aligning expectations with practice. Project leaders might also consider adjusting the practice to minimize access problems, such as by constructing licensing agreements to ensure downstream affordability of the discoveries for underserved populations (AUTM 2007).

### REASONABLE EXPECTATIONS

The final consideration is grounded in the ethical principle of respect for persons and asks whether the ownership practice is consistent with the reasonable expectations of citizen scientists that the project itself has generated. An ownership practice that does not satisfy this condition demonstrates disrespect by failing to make good on promises made or strongly indicated.

There are multiple sources for citizen scientists’ expectations about ownership practices. These include (but are not limited to) prior experiences participating in traditional and citizen science research, conversations with project leaders and non-leaders, and implicit and explicit promises made by the project. To promote analytical feasibility, this consideration focuses solely on sources over which the project has control and can be observed or experienced by affected participants.

First, expectations can arise as a result of project features. For example, if registration for a PersonalPRS challenge includes identification of team members interested in coauthorship of relevant publications, this feature can lead those team members to believe that they might be invited to coauthor relevant publications. Second, expectations can arise as a result of the project’s past actions. If participants in three previous PersonalPRS challenges coauthored publications reporting their findings, participants in the fourth challenge might expect an opportunity to do the same. Participants in the annual Genes in Space contest, which was cofounded by Boeing, might have such an expectation. Every finalist since 2019 has published their proposal, and every winner from 2015 to 2018 has published their experimental results in scientific journals (Genes in Space n.d.a,b). Third, expectations can arise as a result of project communications. If materials advertising a challenge note that benefits of participation include “publication opportunities,” challenge participants will expect those opportunities to be provided. Similar circumstances are presented by the citizen science online game Eterna, which highlights the opportunity for players to “[c]ollaborate on papers for scientific peer review;” several players are lead authors of Eterna’s scientific publications (Eterna n.d.a,b).

To qualify for consideration, however, it is not sufficient for citizen scientists to have expectations that an ownership practice will be implemented in a particular way. The expectation must also be reasonable. Although no formula exists for this determination, some basic principles can be identified. First, it is reasonable for citizen scientists to expect that projects will generally do as they say. Thus, expectations arising from project policies, protocols, and consent forms, as well as other unambiguous written communications with citizen scientists, are likely to be judged reasonable. Second, it is reasonable to expect that projects will comply with their legal obligations. Third, it is not reasonable to expect that projects will act in ways that make it impossible for them to achieve their scientific aims, undermine the integrity or value of their activities, or introduce likely, serious harms to people, communities, or the environment.

When citizen scientists have reasonable expectations regarding a specific ownership practice, the question is whether the practice is consistent with those expectations. If there is a misalignment and it was not caused by unforeseen events, the ethical foundation of the ownership practice is weakened (Woolley et al. 2016). Publication of an article coauthored by one, but not all, participants in a PersonalPRS challenge may seem at odds with promises of publication opportunities, but the other participants might not have been interested or able to satisfy criteria for authorship in scientific journals (Smith, Bélisle-Pipon, and Resnik 2019). In that case, alignment would be improved by clarifying that participants interested in publication will need to satisfy applicable authorship criteria. This example suggests a concrete way for projects to preempt debate about the reasonableness of claimed expectations related to specific ownership practices: clearly articulate how such practices will, and will not, be implemented.

## DISCUSSION

Our proposed framework is intended to be a guide for ethical evaluation of biomedical citizen science ownership practices. It was constructed at a level of generality to potentially be useful to projects regardless of size, setting, objective, or design. In its application, however, context matters. As observed by Cooper and colleagues in their helpful explication of ethical issues relevant to data governance decisions in citizen science, “what arises as an ethical issue and appropriate solution in one project might not in another almost-identical project.” (Cooper, Rasmussen, and Jones 2021, p. 4). Here, also, careful attention must be paid to the details not only of the ownership practice under consideration but how it has or will be operationalized by a project, taking into account its specific features and scientific, organizational, and sociocultural circumstances.

The framework is systematic because it requires examination of each of the four considerations. It is comprehensive because it calls for examination of the various features and implications of an ownership practice from multiple perspectives. But the framework does not purport to be exhaustive. Rather, it leaves open the possibility that, in a particular case, other ethical considerations might require attention. As one example, it may be necessary to consider whether the ownership practice creates, exacerbates, or perpetuates inequities beyond the project that is considering adopting it.

One limitation of the framework’s utility in the biomedical citizen science space is that it will not be relevant to self-experimentation and other n-of-1 activities given that they do not usually present situations where there are multiple interests in or claims to outputs to resolve. Of course, this limitation assumes that such activities qualify as citizen science in the first place. We believe that they can, even if those engaged in n-of-1 activities rarely describe their work as citizen science (Wiggins and Wilbanks 2019).

A different question, raised earlier, is procedural: who is responsible for the analysis and its outcome? Asked differently, whose judgments count? External reviewers knowledgeable about citizen science are well suited for evaluating ownership practices because they are not personally invested in the outcome, but as explained above, external review is not always available to projects. In such cases, project leaders should conduct or oversee the evaluation. Consistent with the citizen science emphasis on fair treatment of participants, it seems reasonable to assume that project leaders will usually handle the evaluation with care. But to promote accountability in the event they do not, leaders should commit in advance to making their analysis available to non-leaders. Of course, some citizen science projects are conducted collaboratively by communities and might not have designated leaders (ECSA 2020). In such instances, all willing participants should be involved or, when this is not practical, representatives should be identified according to processes consistent with project values.

Even when leaders are recognized, they still might want to invite the input of non-leaders on analysis of the four considerations—and potentially others. Indeed, project leaders might feel compelled to do so if, for example, the project’s ownership practices were originally co-created with non-leaders. While soliciting broad input can be helpful and in some cases might be obligatory, leaders should resist any temptation to adopt analytical shortcuts or defaults that would judge a project’s ownership practice as ethically permissible so long as it is endorsed by the project’s non-leader citizen scientists. Our framework describes one way to help guard against unfair outcomes and otherwise increase confidence that judgments about the ethical soundness of ownership practices in biomedical citizen science are systematic, comprehensive, and take into account its singular features.

## Figures and Tables

**Table 1:** Select types* and examples of biomedical citizen science projects.

TYPE	EXAMPLE	REFERENCES
Games: online puzzles that crowdsource solutions to scientific questions through game play	Foldlt; Eterna	Cooper et al. 2010; Lee et al. 2014
Platforms: online platforms for crowdsourcing data collection and interpretation and/or facilitating scientific collaboration	openSNP; Just One Giant Lab (JOGL)	Greshake et al. 2014; JOGL 2022
Community biology: research, exploration, and technology development conducted in non-traditional, community-based settings (e.g., community laboratories)	Open Insulin Project	Gallegos et al. 2018
Biohacking: research, exploration, and technology development conducted in private settings (e.g., home laboratories)	Home genetic engineering	Pearlman 2019
Patient-driven research: research, exploration, and technology development designed, conducted, and/or led by patients, including self-research	DIYAPS OPEN project; PatientsLikeMe lithium self-research	O’Donnell et al. 2019; Wicks et al. 2011
Health hacking: intensive tracking of personal health measurements to monitor or improve health or wellness	Quantified Self “blood tester” study	Grant, Wolf, and Nebeker 2019

* Types are neither exhaustive nor mutually exclusive and are provided with the caveats that terminology and definitions in the biomedical citizen science domain are not settled (Trejo et al. 2021) and multiple typologies to describe biomedical citizen science activities have been described (Guerrini and Contreras 2020).

**Table 2:** The 4Rs: Ethical considerations for assessing ownership practices in citizen science.

CONSIDERATION	KEY QUESTION	ANALYTICAL FOCUS	PRIMARY PRINCIPLE
Reciprocal treatment	Does the practice meet minimum criteria for a fair exchange for citizen scientists given their individual inputs?	Individual inputs	Justice
Relative treatment	Is the practice too generous to some citizen scientists from the perspective of the project’s other citizen scientists given meaningful differences in their individual inputs?	Comparative inputs	Justice
Risk-benefit assessment	Are the anticipated risks and benefits of the practice acceptable?	Consequences	Beneficence
Reasonable expectations	Is the practice aligned with the reasonable expectations of citizen scientists generated by the project?	Project features and activities	Respect for persons
